# Changes in Identity after Aphasic Stroke: Implications for Primary Care

**DOI:** 10.1155/2015/970345

**Published:** 2015-01-21

**Authors:** Benjamin Musser, Joanne Wilkinson, Thomas Gilbert, Barbara G. Bokhour

**Affiliations:** ^1^Department of Emergency Medicine, Los Angeles County-USC, Los Angeles, CA 90033, USA; ^2^Department of Family Medicine, Boston University School of Medicine, Boston, MA 02118, USA; ^3^Department of Community Health Sciences, Boston University School of Public Health, Boston, MA 02118, USA; ^4^Department of Health Policy and Management, Boston University School of Public Health, Boston, MA 02118, USA; ^5^Center for Healthcare Organization and Implementation Research, ENRM Veterans Affairs Hospital, Bedford, MA 01730, USA

## Abstract

*Background.* Stroke survivors with aphasia experience difficulty associated with their communication disorder. While much has been written about aphasia's impacts on partners/family, we lack data regarding the psychosocial adjustment of aphasic stroke survivors, with a paucity of data from the patients themselves. *Methods.* Qualitative study of lived experiences of individuals with poststroke aphasia. Each of the stroke survivors with aphasia completed 3-4 semistructured interviews. In most cases, patients' partners jointly participated in interviews, which were transcribed and analyzed using techniques derived from grounded theory. *Results.* 12 patients were interviewed, with the total of 45 interviews over 18 months. Themes included poststroke changes in patients' relationships and identities, which were altered across several domains including occupational identity, relationship and family roles, and social identity. While all these domains were impacted by aphasia, the impact varied over time. *Conclusion.* Despite the challenges of interviewing individuals with aphasia, we explored aphasia's impacts on how individuals experience their identity and develop new identities months and years after stroke. This data has important implications for primary care of patients with aphasia, including the importance of the long-term primary care relationship in supporting psychosocial adjustment to life after aphasic stroke.

## 1. Background

Stroke is a common cause of morbidity and disability in the United States [1]. Stroke is also the most common cause of aphasia [2, 3], a communication disorder characterized by impairments in the ability to produce/comprehend language. Aphasia has become more visible in recent years as national media have profiled veterans and politicians with traumatic brain injuries [4, 5]. Approximately 80,000 stroke survivors are diagnosed with aphasia each year, and over one million Americans currently live with poststroke aphasia [6]. A common type is expressive aphasia [7], the inability to fluently express one's thoughts while understanding the language of the other person, though individuals with aphasia may also have impairments related to language comprehension. Aphasia type is related to which brain areas were damaged by the stroke. It is important to note that stroke survivors may experience varying degrees and severity of aphasia and that some, but not all, cases of acute poststroke aphasia resolve [6]. Treatment for aphasia includes speech-language therapy, as well as teaching the patient and their family members coping mechanisms and strategies for adapting to life with some degree of communication impairment [6].

Stroke survivors with expressive aphasia experience difficulties navigating their activities of daily living [8, 9]. The literature shows that adjustment to poststroke life with aphasia can be complex and multifaceted [10–14]. While some studies have examined the response of partners [15–18] and families [19, 20] to a patient's aphasia, fewer have explored the impact of aphasia by talking to the patient themselves [21–24] though there is a small amount of literature suggesting that support groups and other social interactions with other aphasia patients can aid in the process of incorporating aphasia into one's identity [25]. In addition, though literature has explored the unique difficulties faced by younger stroke survivors [26], such as childcare/parenting and employment, most of this literature has focused on the return to work [27], rather than personal identity. In addition, the literature related to this population is fragmented, and we lack consistent evidence related to the social needs, experiences, and identities of younger patients with poststroke aphasia [28].

Much has been written about adjustment to and self-concept related to chronic illness [29–31]. Adjustment to aphasia also involves the challenge of living with impairments in communication and the responses those impairments evoke in others [32]. We wondered how aphasia-related changes in relationships might impact other domains of stroke survivors' lives and whether the perception of these challenges and their impact might be different if they were reported by the individuals themselves, particularly to an interviewer with similar speech difficulties. With this in mind, we undertook a qualitative study with the aim of understanding the lived experience of individuals with aphasia and the trajectory of the effect on their social identities, from primarily their own perspectives (and including input and amplification by their partners) in the months and years after their stroke. Though the literature related to younger patients (aged 65 and under) with aphasia has focused on the return to work, we were interested in other, nonwork related aspects of identity, though we recognize that one's identity as a worker also influences their overall self-concept. In the analysis, we noted themes of impact on multiple aspects of identity, such as professional identity, familial identity, social identity, and the interactions of these various components of identity [33, 34]. Sociological theory posits that individuals who hold multiple identities (i.e., worker, neighbor, friend, parent, spouse, etc.) may be more resilient [35]. In this study, we explored the potential for resiliency among patients with aphasia.

## 2. Methods

### 2.1. Participants

We recruited two cohorts for this project. The first (cohort 1) was patients who had recently experienced a stroke resulting in aphasia and were in-patients in a Boston-area rehabilitation hospital at the time of recruitment. These patients were in-patients at the time of the initial interview and were followed for an average of 18 months, completing additional interviews of the course of this time period. These participants had to comprehend questions and be able to indicate their consent, have significant expressive aphasia, and live within the local urban/exurban area for the purposes of follow-up. Participants were excluded if they were considered to have a poor prognosis by the rehab physician (i.e., patients who the physiatrist felt would likely not live the 18–24 months to engage in study follow-up). The second (cohort 2) was participants who had their stroke more than 5 years prior to the study and were involved in a local aphasia support group. Both cohorts of participants were approached by co-PI TG, a retired physician who himself had aphasia secondary to a stroke in 1999. (TG has made a significant recovery and is able to communicate effectively, though he still faces some language difficulty.) Of the original 14 individuals approached, most were enthusiastic about participating. Three individuals ultimately declined after reviewing the study materials further with their partner. One participant from cohort 1 was lost to follow-up after one year, moving several times. For both cohorts, we continued recruiting until saturation [36] (the point at which novel themes are no longer being generated from new data) had been reached. The demographics of the participants (Tables 1 and 2) reveal that they were relatively young both at the time of their stroke (43–55 years old) and during their involvement in the study (46–71 years old). This selection was not intended by the investigators, and while these demographics may differ from the usual profile of participants with aphasia, it provides an opportunity to investigate multiple facets of identity since all were in the workforce when they had their stroke.

### 2.2. Data Collection

TG conducted semistructured interviews with patients and their partners, if applicable. (Though the presence of a partner was not a requirement for participation, in our final sample, all but one patient were married or in a long-term relationship.) Patients and their partners were asked whether they desired partner presence at the interview; all participants in long-term relationships elected to have their partners participate. All of the participant interviews were done by co-PI TG. In addition, co-PI JW interviewed TG and his spouse regarding their experiences; these interviews were also included in our data. As a stroke survivor with aphasia, he was able to describe his own experiences just as the other participants did, and because we allowed the content of the interviews to determine the themes of the study, we were confident that these interviews did not add any biased or prompted information. Participants and their spouses consented to being audio/video-recorded during the interviews, and the majority of interviews involved both the participant and their partner.

For participants in cohort 1, the following general topics were covered: (1) their perception of their progress and feelings about their current limitations and progress; (2) their mood; (3) activities they were engaged in and things that were important to them; and (4) the relationship between the participant and their partner, if applicable. For participants in cohort 2, the general topics were very similar, but participants were also asked to reflect back on how they felt about the issues above after their stroke and how their feelings about those issues had changed, if at all, over the years. Open-ended questions were followed by prompts if needed, with flexibility to follow up on any new topics introduced by participants. In designing the interview guide, care was taken to keep questions as open-ended as possible and to allow participants the chance to tell their stories.

Follow up interviews were conducted at approximately 3, 6, 12, and 18 months after the initial interview and were scheduled according to participant preference and convenience. We interviewed a total of twelve participants in forty-five separate interviews; six of them were interviewed at least five times, and the other six were interviewed at least once, generating 653 single-spaced pages of transcription. Participant demographics are available in Table 1. Participants had varied abilities to express themselves fluently; in some cases, they responded completely and fluently to the question posed, and in some cases, they responded in a more limited way, often with some content filled in by their partner. By having partners present for most interviews and rechecking certain themes and statements over time as participants' speech improved, we felt confident that the data obtained from the interviews were a valid representation of what participants sought to express.

All the interviews were transcribed by the same transcriptionist, a Ph.D. with training in anthropology who used ethnographic methods to accurately transcribe every utterance of the participants with aphasia [37].

### 2.3. Data Analysis

We conducted analyses using techniques informed by grounded theory methodology [36], a qualitative analysis method which emphasizes allowing analysts to identify themes as they emerge. The interviews were initially coded by at least two members of the research team (JW, TG, CD, JYK, RR, BM, and LBW) line by line with one- or two-word summaries of the content. Next, those codes were reviewed jointly by the coders and grouped into second-level codes (categorizing/defining them more broadly). Third, the second-level codes were reviewed and grouped as broader themes were identified from the data. As we identified themes from the data using an iterative process, we periodically went back to the transcripts to confirm their meaning. Using this method, we were able to make slight adjustments to the interview guides (for both groups) as we went on recruiting subjects, to allow us to explore certain ideas in more depth. Each step in this process was reviewed by at least two researchers.

Given the limitations in language from participants (particularly those early in their recovery), our analysis relied in part on the conversational collaboration of their partner. In some cases, partners merely clarified or amplified what participants were expressing in the interview; in others, partners introduced new ideas or topics that had not been raised by the participant initially. We coded these instances differently during the analysis.

The project was IRB-approved; all initials and names referring to participants in this paper are pseudonyms.

## 3. Results

In analyzing our data, changes in identity emerged as an important theme, identified by nearly all participants. We found three additional themes related to identity change in different domains, including occupational identity, relationship/family roles, and social identity.

### 3.1. Occupational Identity

All the participants were employed prior to their stroke, and most were working full-time in a professional capacity (e.g., accountants, professors, and lawyers). None were able to return to their previous occupation. Participants spoke about the difficulty they had accepting this and their struggle to find some kind of work they could do.

Participant J. (Table 3) narrated the difficulty he had accepting that he could not return to work, describing his experience of driving to his office repeatedly and trying to engage in his previous occupation. Participant S., whose stroke was more recent, was still trying to come to terms with his options for employment and income. Both participants express some of the sense of displacement they felt when they were abruptly forced to retire from their professions.

Many participants, once they had received a certain amount of rehabilitation, were eager to engage in some kind of meaningful activity. Several became hospital volunteers or peer visitors to other stroke patients; they were able to draw on their own experiences in a way that they felt would be helpful to others. One participant, B., who had previously worked as a computer programmer, responded enthusiastically to the subject of volunteering at the hospital (see Table 3). B. and his partner framed his volunteer work in terms of an earlier aspiration to be a physician. This reclaiming of an occupational identity helped him to embrace a sense of meaning in his daily activities.

Other participants contrasted their current volunteer activities with what they might have been doing if they had not had the stroke. In Table 3, J. describes a typical week for him and then also muses about what his occupational activities would be had he not had the stroke. He acknowledges that, even without the stroke, his occupational identity could have changed and grown as he did. Both B. and J. describe their new activities as satisfying: in B.'s case, a purposeful activity that for him partially fulfills an earlier dream of working in health care and, in J.'s case, a variety of activities that are different from his prior work, with the realization that his prior work might also have changed with time, with or without the stroke.

### 3.2. Relationships and Family Roles

While the changes in occupational identity were often individually experienced, the shifts in relationships and family roles were often jointly negotiated by the participant and other people, their partner and/or children. Participants and their partners spoke of changes in the dynamic of their relationship as the result of aphasia. In some cases, the balance of responsibilities in the relationship shifted. While the participants spoke about the impact of aphasia on their occupation, in Table 4 J.'s wife characterized the experience in terms of this shift in balance and roles. She acknowledged feeling overwhelmed by the change. However, not all partners perceived this change as negative. T.'s wife talked about the satisfaction of discovering new skills (Table 4).

Some participants spoke of changes in other roles in the family, for example, as a parent. M. routinely made snacks for her children and the neighborhood children after school and hosted them at her house. She spoke about losing that activity in Table 4, in part because her children felt self-conscious about bringing their friends home after her stroke. Her description of how she misses the “house that was always happening” is also a loss of part of her identity, as the neighborhood mom whose home was always open and ready to serve as a community gathering place.

Another participant, V., who had coached his son's football team ultimately returned to that activity as a way to remain connected with his son. He did this even though his aphasia still prevented him from conversing fluently with the players. V.'s participation in football allowed him to sustain his identity as a coach; it is interesting to note that, in this setting (working with adolescent boys), his cursing (likely a consequence of his word finding difficulties) was viewed with a positive spin and did not impede his ability to be effective and well liked by players.

### 3.3. Social Identity

Participants and their partners also talked about the changes they experienced in their sense of themselves in their broader social lives: their level of independence and their ability to meet and talk to new people. They also discussed the impact of the aphasia on their friendships and social support, especially in terms of the partner. In Table 5, V.'s wife notes his difficulty meeting and talking to people. She describes him as friendly and talkative before the stroke, a personal style that she feels has since changed. Here, she reflects that his aphasia makes it much harder for him to relate to people in the way he did before and results in his withdrawing more at social events and enjoying them less. Previously, V. knew himself as someone who enjoys being around others; now, he is more self-conscious and avoids situations where he might have to introduce himself or talk to acquaintances. As V.'s wife notes, his stroke has changed not his ability to interact with others, but the more profound change is in V. himself: his desire to be social and his social identity.

Other participants noted these challenges as well, although in some cases they were pleasantly surprised with their interactions with new people. B. and his partner (Table 5) found a novel way to enjoy an old leisure activity (visiting a casino) and described the adventure in a positive way.

Participants and their partners expressed a range of experiences regarding friends and support from their social circle. Their responses to those experiences helped to shape some of their new social identity. For example, M., a mother who was frequently involved in neighborhood activities, speaks (Table 5) about her ability to function again as a member of that circle.

In contrast, B.'s partner (Table 5) perceived that their friends and acquaintances disappeared after the stroke. Still another group of participants and partners felt that while not all of their friends were helpful, they received support from unanticipated places. In Table 5, T. and his wife discuss this phenomenon. This couple (several years after stroke at the time of the interview) collaborated to find a way to understand and view the responses from their social circle in a way that made sense to them and affirmed their social identity.

While all participants agreed that the experience of having a stroke and having aphasia impacted their social lives, the perceptions of that impact could be very different depending on the participant and their partner.

## 4. Discussion

Our interviews with patients and their partners revealed how aphasia can cause changes in three aspects of identity: occupational, family, and social aspects. Participants described the impact of their aphasia on the way they perceived themselves and others perceived them in all of these settings. Not all of these changes were described as negative, though many were framed as challenging initially until the participant found a way to create a new identity for themselves.

Much has been written about the impact of chronic and/or catastrophic illness on identity [29–31, 38, 39]. In particular, the challenges of identity reformation after stroke and traumatic brain injury have been described [40–42]. Mattingly [43] and others describe the patient's important work as learning to narrate a new “story” for themselves that incorporates their particular limitations after injury. This work of creating a new story was reflected by participants in our study as they talked about changes in identity over time. As they described, the work of recovery from aphasic stroke involves creating a new narrative of themselves and who they are. Patients with aphasia may have less audience and external input into their narratives, given their limitations in communication. This may mean that spouses and loved ones play a significant role in helping the patient verbalize the narrative to the external world, potentially editing aspects of their new narratives as they are shared. This perspective is important for primary care providers to understand as they care for patients with aphasia.

For the participants in our study, language and communication were an important part of their identity: as professionals, as parents, as friends, and as spouses. Impairments in these areas forced them to recreate or shift their identities; this process was dependent on several factors. These included previous interests they may have held, the severity of their aphasia [44], the support of their spouse or partner [45, 46], their relationships with friends, and their ability to frame their experiences in new and adaptive ways. This last trait is particularly likely to be impacted by depression, which is extremely common after aphasic stroke [44, 47]. Many participants in our study spoke about mood changes as they adjusted to their aphasia. From the perspective of the individuals we interviewed, their mood was closely tied to their ability to adjust/adapt to change, reframing their identity shift positively. B., for example, described an improvement in mood when he decided to become a peer volunteer at the hospital, in part because he framed that decision as a way to realize an earlier dream of becoming a physician to “help people.”

In many cases, neurologists and rehabilitation professionals are involved with stroke patients for a relatively short time, under 6 months, and therefore are less well positioned to be able to facilitate the work of creating a new narrative. However, primary care physicians involved with the patient over time are more able to provide feedback, coaching, and appropriate treatment for depression, if applicable, all of which may help the patient to move forward in the formation of their new identity. Recognizing patients' struggles with their identities and in creating a new narrative may help in fostering recovery for these patients.

Our study had several limitations. We recruited a sample of patients who were overall a group of upper/middle class professionals, well-educated, with strong family and spousal support. These individuals, recruited using a purposive sampling strategy, were not representative of the all patients, which skews our results. However, it should be noted that people in relatively advantaged social positions faced great challenge in adjusting to the impacts of aphasia upon their social identities. Though further research is of course needed to clarify the impact of socioeconomic status (SES) on aphasia support and adjustment, we theorize that less access to social and economic resources may magnify many of the challenges discussed by our participants. (We plan, in the future, to explore the lived experience of individuals with aphasia who are of low income, who are members or minority population, and who may not have family or partners involved in their care.) In addition, our decision to use a purposive sample for cohort 2, recruiting individuals from a community-based aphasia support group, also presents a limitation, in the fact that individuals who participated in this cohort inherently have more community-based supports than individuals who do not attend support groups. Furthermore, participation in support groups or other group-based activities is known to positively impact social adjustment to poststroke life [48]; our participants therefore had advantages that might not be applicable to all patients, perhaps presenting an overly positive picture. Patients who are not involved in support groups may have more or additional kinds of challenges that we do not know about; further research is needed with less advantaged populations, as discussed above. Interviewing individuals with aphasia did mean that they were unable to fully express themselves verbally. We were fairly confident that, between the interviewer's familiarity with aphasia and the partner's input, we were able to deduce the intended meaning of the utterances of the aphasic participants when they were unclear. Additionally, our primary interviewer was himself someone with aphasia. While we believe this added to the comfort of the participants and his insight into their life stories, it is also possible that his presence and experiences may have unwittingly influenced the topics covered in the interviews.

Our findings suggest that changes to identity after aphasic stroke span multiple domains and require that patients rebuild their previously held sense of self. This psychological and social task may be an important component to poststroke recovery. Potential future directions of this research include exploring the experiences of patients from different races, classes, and educational and family backgrounds and the development of educational materials and resources for patients with aphasia as they begin the journey to rewrite their life story and move forward with their recovery.

## Figures and Tables

**Table 1:** Subject demographics.

Subject^*^	Age at stroke	Age at time of interview	Years since stroke	Gender
B.	48	52	4	M
P.	50	52	2	M
M.	45	46	2	F
R.	55	56	1	F
V.	51	53	2	M
S.	49	51	2	M
H.	43	71	28	M
C.	52	62	10	M
K.	54	62	8	M
E.	49	57	8	M
J.	49	64	15	M
T.	52	64	12	M

^*^Names have been changed.

**Table 2:** Summary demographics of participants.

Demographic	% (*n*)
Gender	
Female	16.67% (2)
Male	83.33% (10)
Age at stroke	
40–45	16.67% (2)
46–50	41.67% (5)
51–55	41.67% (5)
Current age	
46–50	8.33% (1)
51–55	33.33% (4)
56–60	16.67% (2)
61–65	33.33% (4)
66–70	0% (0)
71–75	8.33% (1)
Years since stroke	
0–5	50% (6)
6–10	25% (3)
11–15	16.67% (2)
25+	8.33% (1)

^*^Names have been changed.

**Table 3:** 

**Participant S [interview was 6 monthsafter stroke]**: You know, is it—you know, like, do I go onto disability for the—my entire life, or what do I do? And that's where I'm at right now.	

**Interviewer**: If you want to come in to, ah, [Hospital], to give some back. **Participant B. [2 years after stroke]**: Yes! Yes! In fact, here, go ahead. **B.'s partner**: Do you want to give back? **B.**: YES!! **Partner**: Do you want to help? **B.**: YES!! YES!! YES!! Yes, because— **Partner**: Well, we'll be able to make that happen. **B.**: Yes! Because—because things will be better.	

**B.'s partner**: B always wanted to be-well, he had seriously considered a career in medicine. And I think he couldn't pass chemistry, it was one-it was something like that. [laughter] **B. [2 years after stroke]:** Yes, but-but now- **B.'s Partner**: You know, by default, you're going to end up in medicine somehow.	

**J. [15 years afterstroke]**: Monday is…Lazarus House. And it's nice, it's—and I've been going there since the beginning. **J.'s wife**: It's a little thrift shop. **J.**: Tuesday, um, it's— **J.'s wife**: The library. **J.**: Wednesday is, um, usually a switch between…I take on Monday, on Wednesday…it's a class at [university]. And then, um, Thursday is—I don't know what it is. **J.'s wife**: [Name of rehab hospital where J works as a peer volunteer.] **J.**: So [hospital], yeah, that's right. Friday is the day off.	

**J.**: But I won—I wonder what it would be like to not have a stroke. What would I do? **J.'s wife**: You'd be busy working. **J.**: I know that. But I wonder what it—I—I'm not working at [company name] now. I'm working someplace else. What would—would that be? Maybe I would—Maybe I give up teaching— **J.'s wife**: Or you're probably working in a startup. **J.**: A startup, maybe a startup? Maybe I'm more—maybe I'm going to help other kids make the break from college, master's degree or whatever into—So I really don't know what I—what I could do. I never knew that.	

**Table 4:** Family identity quotes from participants.

**J.'s wife [15 years afterstroke]**: After 25 years, we switched roles. That's how I look at it, because we were married 25 years, the March—the October before he had the stroke in March. So we basically switched roles. So now I go to work all day and work in corporate America, and come home, and he's waiting at the door for his drink and dinner. (laughter) Where I used to wait for him to come home and have dinner almost ready or something when he walked in the door. A little bit different.	

**J.'s wife**: And because he did everything—I mean he—I might have done all the cooking and the shopping and the buying and the kids, and all that good time stuff, but he did all the financials. I had no clue about what the financial—our finances were or where things were. I had to learn all of that and do that—you know, I just spent one whole day just in an office, just reading things. I mean, I had no clue if he had extra life insurance, if he didn't have life insurance.	

**T.'s wife [11 years afterstroke]**: I think it did bring me new skills. I don't think I was ever in a position to really have to problem solve this way. And so—and there's a lot of satisfaction, being able to figure something out, you know. **T**: Yes.	

**M. [1 year afterstroke]**: But, like, what I want to do for them—…peop—to say, “Hey, come”—the—the boys, “Come on over, they can play in the yard, and then I'll”—because I used to cook for them. And you know—and then they—they were so appreciative.…I don't know, I—I—I miss it. You know, like if the—the, um, house is not, like—like, the kids are always over here. And you know, they've been here, like—you know, it's been—they just don't come, you know. So I—I miss that—that house, that—you know, was always happening, you know.	

**V.'s wife [1 year afterstroke]**: And V. is still one of the coaches for the football team. He's always helped coach the kids' football. So he's still out there on the field, and the kids love it…One of the mothers had said to one of the coaches, she said, I don't know who it is, but my son just loves this one coach. And he's like, oh, that's great, but there's like six or seven of us. Which one is it that he loves so much? And she said, the one that says “shit” all the time. (laughter) He said, oh, that would be V.	

**Table 5:** 

**V.'s wife [9 months afterstroke]**: …like a big cookout, my friends. What normally would be a lot of fun, and just mingling with everybody, and he seems to drop back on wanting to go. He doesn't—or if he does go, he doesn't want to stay for that long, you know, a couple of hours or so and he's fine…And I think it's because of the communication that—I think it's because it is hard for everyone to (inaudible), so they talk to him, the whole bit, everybody loves to see him, and everything. But then, I think he feels left out because he can't contribute, you know, a normal conversation with him.	

**B.'s partner [6 months afterstroke]**: …at the casino we were having a blast…[We] played a couple of slot machines, just hung out for the afternoon. **B:** Yes! … **B.'s partner:** And B. got off the machine and went and sat at the bar. Sat down to a woman who was hell-bent on talking to him. And she kind of finally figured out, OK, there's a—you know, an issue going on here. She saw the cane. I walked up at that moment, told her he had had a stroke. She knew all about it. She was his new best friend. She—she was nice. **B.:** Yes! **B.'s partner:** She asked, you know, exactly how to talk to him, ask him the yes-no questions. And they were there for a good 45 minutes. As far as I was concerned, you had a great day.	

**M. [1 year afterstroke]**: I think that [driving] is going to make a huge difference. I think the boys, um—you know, I'll feel better, like—what—what's my, like, um—being able to help them, you know what I mean? The—the people that help—like, we have so many friends, thank God, that they drive the kids everywhere. So I hope that, you know, I can take their, um, kids, you know. So that will make—big—a huge difference, you know.	

**B.'s partner [2 years afterstroke]**: And the ones that were my true friends, that I thought were in it for the long haul, eighteen, twenty, twenty-five years of friendship, (claps hands), gone. They are so far out of my life now. The phone stopped ringing. Christmas cards stopped. Everything.	

**T.'s wife [11 years afterstroke]**: **…**the thing I observed was I think men, it was very scary for them when he had the stroke. And I—I think they sort of avoided him for a while. You know, like it was catching or something. **T. [11 years afterstroke]**: In general, um, people that were [friends] before the stroke are the same after the stroke. However, and I think this is true for anybody. There are people who don't do wonderfully after the stroke, and other people who do. And you can't predict them ahead of time.	
**T.'s wife**: Well, I think, um—one thing I'd say is that people give you a lot of support, you know, as a caregiver. And it's always surprising who comes out of the woodwork. It's not necessarily the people you think are going to, but I think that helped me a lot. You know, especially the first year when I was home, and I wasn't used to not working. And you know, people would call and cheer us on.	
